# On the chemistry of 1-pyrroline in solution and in the gas phase

**DOI:** 10.1038/s41598-017-08217-1

**Published:** 2017-08-09

**Authors:** Xiaoping Zhang, Konstantin Chingin, Dacai Zhong, Juchao Liang, Yongzhong Ouyang, Huanwen Chen

**Affiliations:** Jiangxi Key Laboratory for Mass Spectrometry and Instrumentation, East China University of Technology, Nanchang, 330013 P.R. China

## Abstract

1-Pyrroline has a highly characteristic odor, which is employed by living organisms for chemical signaling and other purposes, but the mechanism whereby this odor is formed remains poorly understood. Here we used a combination of ambient mass spectrometry (AMS) and nuclear magnetic resonance (NMR) spectroscopy to experimentally address the mechanistic aspects of 1-pyrroline volatility and other controversies regarding the chemistry of this compound. Our results indicate that in solution the volatility of the monomer species is significantly higher than that of the trimer species, and 1-pyrroline is evaporated mainly in its monomer state. Neat 1-pyrroline is essentially the pure trimer and displays ca. 100-fold lower evaporation rate than the monomer state in solution. In the gas-phase the trimer species is irreversibly decomposed into monomer species. Under equilibrium conditions the vapor of 1-pyrroline entirely consists of monomer species. The evaporation rate of 1-pyrroline in water has a step-wise dependence on the solution pH, the abrupt increase in volatility (>1,000-fold) occurring around the pKa value of 1-pyrroline (6.8). The pronounced step-wise dependence of 1-pyrroline volatility around neutral pH may also be an important evolutionary factor allowing living systems to regulate the odor strength from very weak to very strong with minimal efforts.

## Introduction

1-Pyrroline is a natural volatile compound with highly characteristic odor that closely resembles the odor of human semen^1^. 1-Pyrroline is released into the ambient air by insects^2, 3^, presumably, as a component of sex pheromone^4–6^, by plants for signaling and odor mimicking purposes^7–9^, as well as by bacteria during pre-growth phase^10, 11^. 1-Pyrroline is also essential to human olfaction as a primary odor with extremely low detection threshold^1^.

**Figure 1:**
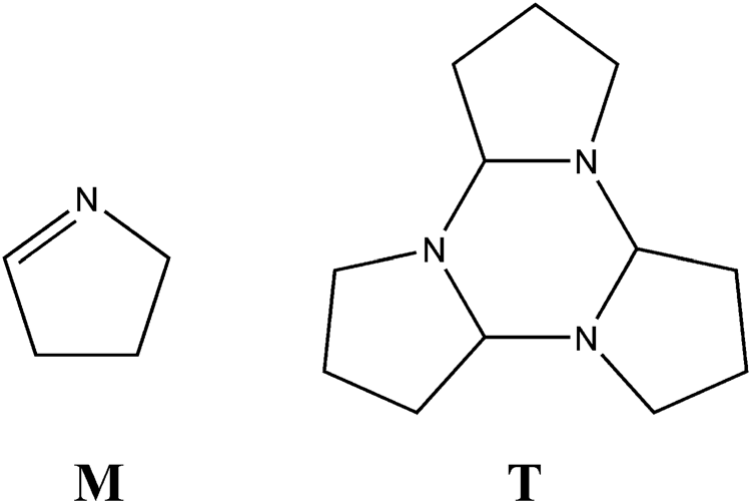
The monomer and the trimer states of 1-pyrroline.

Despite the biological implications of 1-pyrroline odor, the mechanism of its formation remains very scarcely studied^12–14^. The major obstacles include the poor chemical and thermal stability, the lack of commercially available standard and the lack of simple synthesis protocol for 1-pyrroline. Earlier studies indicate that in liquid solution 1-pyrroline is present in equilibrium between its monomer (*M*_*s*_) and trimer (*T*_*s*_) states (Fig. 1), *T*_*s*_ being more thermodynamically stable than *M*_*s*_ at room temperature (ΔG ≈ 2 kcal/mol in DMSO)^13^. In contrast, only the monomer of 1-pyrroline was detected in the gas phase by microwave and infrared analysis, suggesting that the equilibrium concentration of gas-phase monomer (*M*_*g*_) is much higher than that of gas-phase trimer (*T*_*g*_)^13, 15^. However, there are many aspects regarding the chemistry and odor of 1-pyrroline that remain unclear. For example, there is yet no conclusive proof whether the characteristic semen-like odor of 1-pyrroline is caused by *M*_*g*_ or by the trace amounts (undetectable by experiment) of *T*_*g*_. Further, it remains largely unknown whether *M*_*g*_ is preferentially formed via the evaporation of *T*_*s*_ followed by the irreversible decomposition of *T*_*g*_ into *M*_*g*_ or, alternatively, directly via the evaporation of *M*_*s*_. Also, the volatility of 1-pyrroline in solution as well as the effect of chemical matrix on the odor strength of 1-pyrroline has not been studied in detail. In this study we conducted a series of experiments in order to address these questions as well as to clarify other aspects concerning the chemistry of 1-pyrroline in solution and in the gas phase.

## Results

### NMR analysis

**Figure 2:**
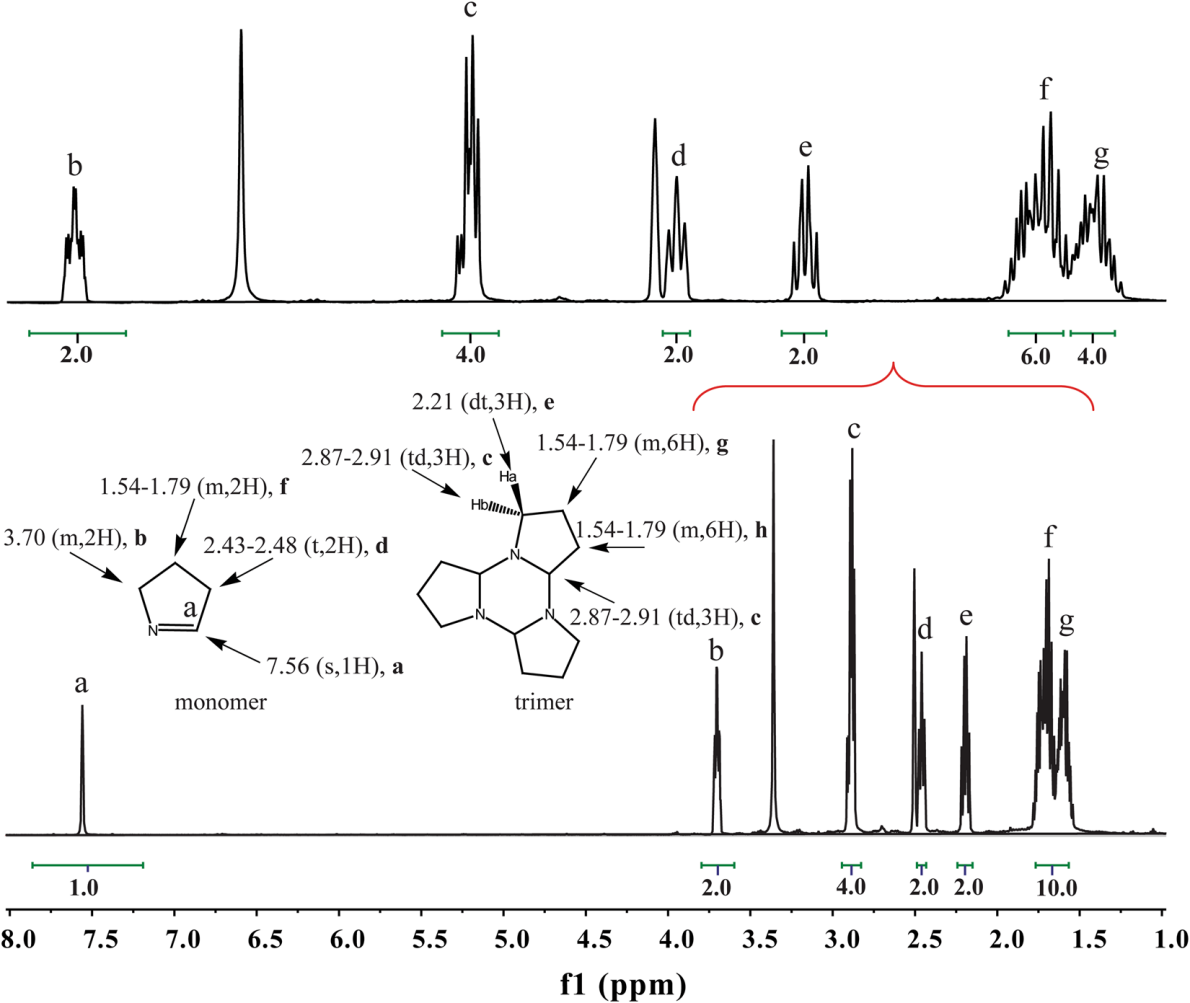
^1^H-NMR spectrum of 1-pyrroline solution in DMSO-*d*_6_ (10,000 ppm) displaying the chemical shift assignments for the monomer and trimer.

The purity of synthesized 1-pyrroline sample was confirmed by NMR analysis. Fresh 1-pyrroline was diluted in different deuterated solvents, such as DMSO-*d*_6_, CDCl_3_, and D_2_O. No other signals could be detected apart from those belonging to 1-pyrroline monomer, trimer, and corresponding deuterated solvent (Figs. 2, S1, and S2).

The NMR spectral data for both the monomer and trimer obtained from 1-pyrroline sample with different concentrations and solvents indicate equilibration of the monomer with its trimer form on the timescale of dissolution, which is in agreement with earlier studies^13^. This equilibrium is concentration dependent^16^. The spectral data for the both monomer and trimer of prepared 1-pyrroline in 100-fold diluted solution (Fig. 2) are as follows: Monomer: ^1^H NMR (500 MHz, DMSO-*d*_6_) δ 7.56 (s, 1H), 3.70 (m, 2H), 2.43–2.48 (t, 2H), 1.54–1.78 (m, 2H); Trimer: ^1^H NMR (500 MHz, DMSO-*d*_6_) δ 1.54–1.79 (m, 12H), 2.87–2.91 (td, 6H), 2.21 (dt, 3H). Monomer: ^13^C NMR (126 MHz, DMSO-*d*_6_) δ 166.5, 60.7, 36.4, 20.2 ppm; Trimer: ^13^C NMR (126 MHz, DMSO-*d*_6_) δ 81.4, 45.3, 27.5, 19.9 ppm. Only trimer signals were observed in undiluted neat sample, indicating that the neat material is essentially purely trimer. This is consistent with the earlier observations^13, 17^.

### *M*_*s*_/*T*_*s*_ equilibrium

**Figure 3:**
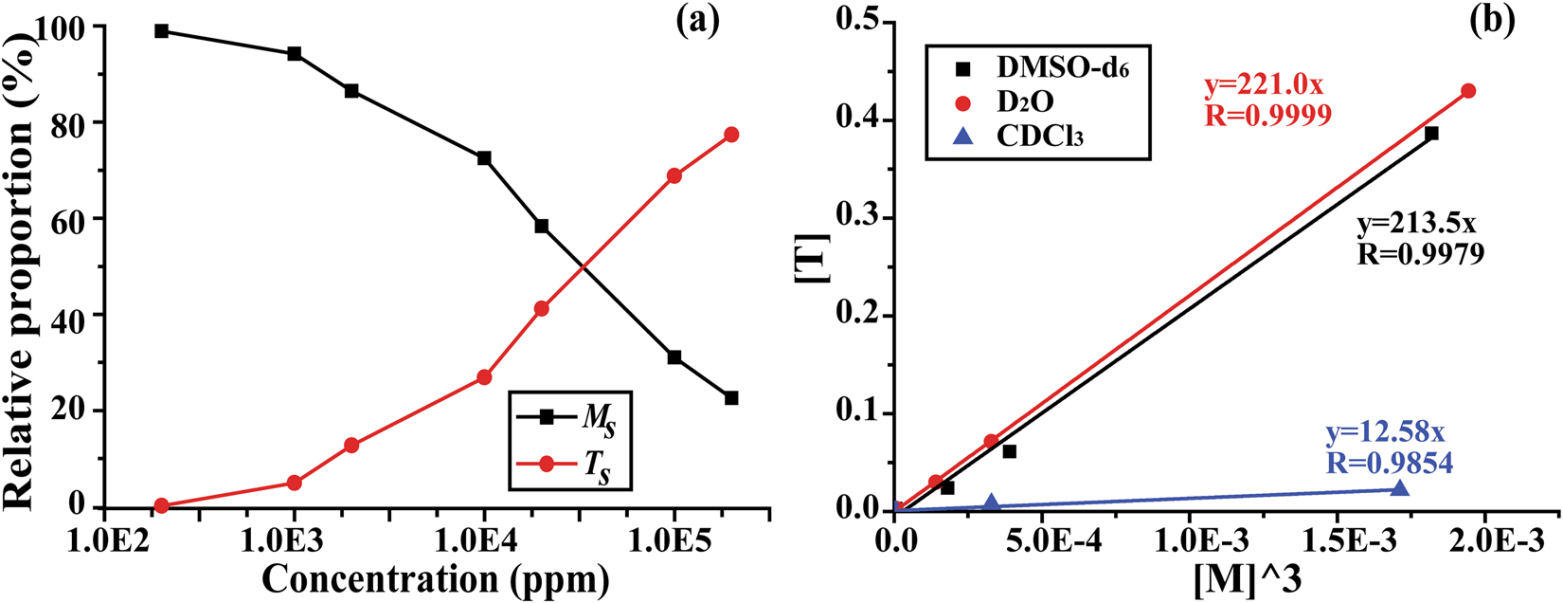
The relative proportion of *T*_*s*_ and *M*_*s*_ in DMSO-*d*_6_ solution as a function of 1-pyrroline concentration (**a**), and the linear relation between [*T*_*s*_] and [*M*_*s*_]^3^ in different solvents (**b**).

Fig. 3(a) shows the proportion of [*T*_*s*_] and [*M*_*s*_] in diluted 1-pyrroline solution as a function of total 1-pyrroline concentration in DMSO-*d*_6_ derived from the ^1^H NMR spectrum by integrating the relative abundance of monomer and trimer signals. At low concentration (<1,000 ppm) 1-pyrroline exists essentially in *M*_*s*_ state. As the concentration of 1-pyrroline grows, the share of *M*_*s*_ is decreased and the share of *T*_*s*_ is proportionally increased up to the level at which only *T*_*s*_ is observed (neat 1-pyrroline). In a similar way, the relative proportion of [*T*_*s*_] and [*M*_*s*_] in diluted 1-pyrroline solution as a function of total 1-pyrroline concentration was also derived in CDCl_3_ and D_2_O (Fig. S3). Fig. 3(b) shows the relationship [*T*_*s*_] vs [*M*_*s*_]^3^ at different total concentration of 1-pyrroline in three solvents: D_2_O, CDCl_3_, DMSO-*d*_6_. The reciprocal of linear slope corresponds to the equilibrium constant *K*_*eq*_^(s)^ = [*M*_*s*_]^3^/[*T*_*s*_]. The high linearity indicates that the equilibrium between *T*_*s*_ and *M*_*s*_ obeys third-order kinetics in a wide concentration range. The equilibrium kinetics has a very high speed and is readily re-established upon dilution, as has been shown by earlier studies^13^. The results indicate that at 300 K *T*_*s*_ is more thermodynamically stable than *M*_*s*_. From our results we calculated that the free energy of conversion from *T*_*s*_ to *M*_*s*_ in DMSO-*d*_6_ is equal to ΔG = −RT ln(*K*_*eq*_^(s)^) ≈ 3.20 kcal/mol, which is higher than the earlier estimate for ΔG ≈ 2 kcal/mol obtained for 1-pyrroline in DMSO-*d*_6_ by Baker *et al*.^13^. Our results therefore suggest that the relative stability of *T*_*s*_ in solution is higher than that based on earlier estimates. The free energy difference between *M*_*s*_ and *T*_*s*_ in D_2_O and CDCl_3_ was ΔG ≈ 3.2 kcal/mol and ΔG ≈ 1.5 kcal/mol accordingly.

### Ambient MS analysis

The purity of synthesized 1-pyrroline sample was additionally confirmed by the ambient atmospheric pressure chemical ionization mass spectrometry (APCI-MS) analysis of vapor phase (Fig. S4). Only the presence of 1-pyrroline monomer could be detected in the form of a protonated cation (*m*/*z* 70). No trace of the trimer species could be detected even from the pure undiluted sample. If present in the vapor phase, 1-pyrroline trimer would have been observed as a protonated cation at *m*/*z* 208. The lack of *T*_*g*_ signal in APCI-MS of 1-pyrroline vapor is consistent with the results of earlier microwave spectroscopy experiments that also indicated the lack of *T*_*g*_ in 1-pyrroline vapor^15^. Consistent with the results of NMR analysis (Figs. 2, S1, S2), our APCI-MS analysis also indicate the high purity of the synthesized 1-pyrroline sample. No trace of possible volatile contaminants, such as protonated 4-aminobutanal (*m*/*z* 88) or protonated pyrrolidine (*m*/*z* 72), was revealed in the mass spectrum (Fig. S4).

Fig. S5 shows the response range of 1-pyrroline APCI-MS signal (*m*/*z* 70) as a function of 1-pyrroline concentration in water solution in the range from 10 to 400 ppb and in working DMSO solution in the range from 1 to 100 ppb. Both plots reveal high linearity between signal intensity and 1-pyrroline concentration in the low concentration range. In order to measure the concentration of 1-pyrroline in concentrated solutions, the corresponding samples were first diluted down to the linear range (Fig. S5), and the original concentration in the concentrated solution was calculated from the signal intensity in the linear range and the known dilution rate.

### Evaporative loss of 1-pyrroline in water

**Figure 4:**
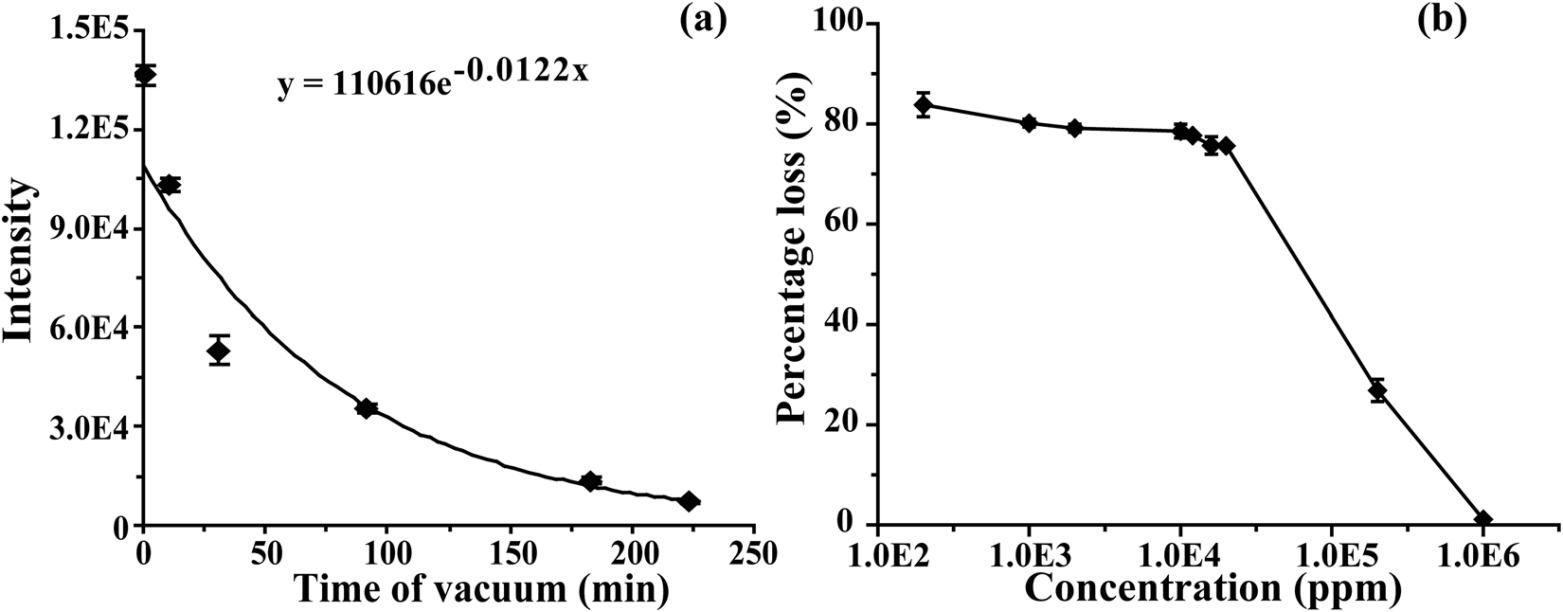
Evaporative loss of 1-pyrroline from aqueous solution with a concentration of 200 ppb as a function of vacuum evacuation time (**a**), and the evaporative loss of 1-pyrroline from DMSO solution at a fixed evacuation time (30 min) as a function of concentration (**b**) measured by APCI-MS analysis.

Evaporative loss of 1-pyrroline from 4 mL solution in water with a concentration of 10 ppm (pH 6.8) as a function of vacuum (−0.1 MPa) evacuation time was measured by APCI-MS analysis (Fig. 4). The sample was accordingly diluted to 200 ppb before the analysis to the linear response range of the instrument (Fig. S5a). The derived loss of 1-pyrroline was ca. 60% per 30 min. Water loss was ca. 12% weight per 30 min and was replenished every 30 min to the original level. Based on the exponential fit of evaporation kinetics ([*C*_*s*_] = [*C*_*s*_]_0_exp(−α*t*)), the evaporation rate (α) of 1-pyrroline in water is estimated to be ca. 7 times higher than the evaporation rate of water under the same conditions. No evaporative loss of 1-pyrroline was observed over the same time period in the reference experiment when the bottle was open under air without vacuum evacuation.

### The effect of pH value

**Figure 5:**
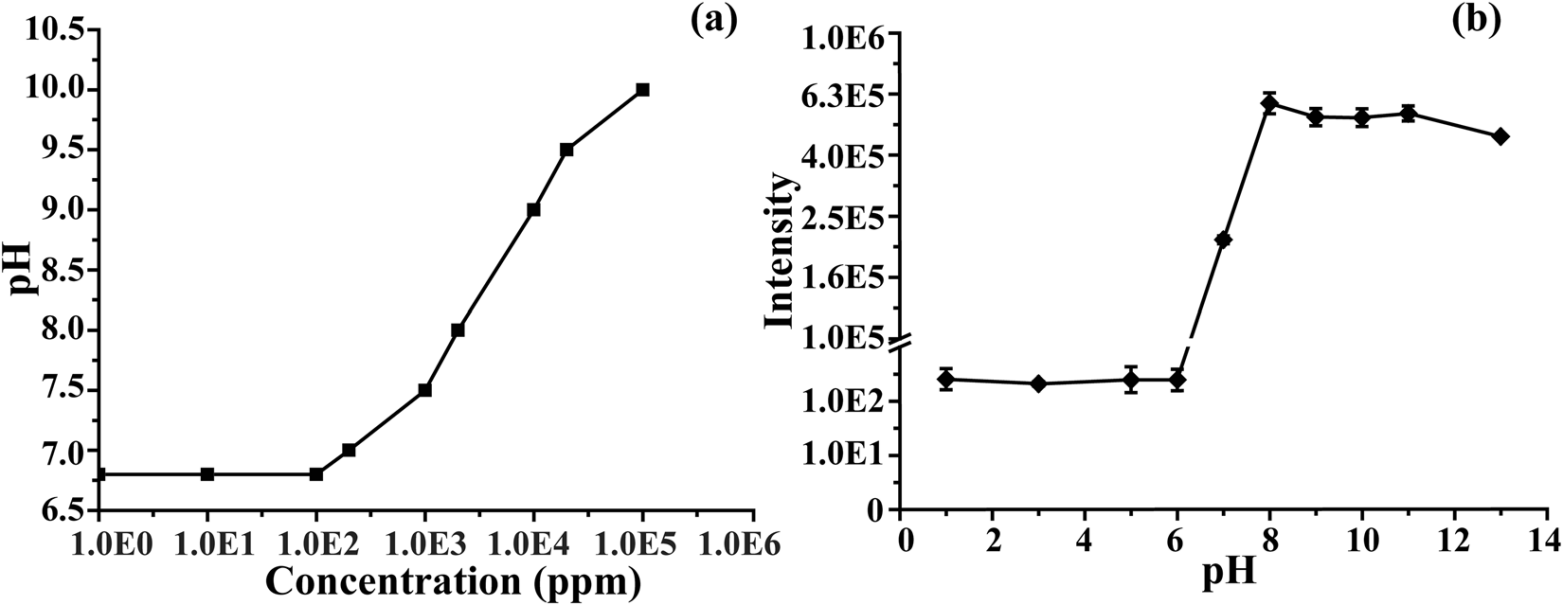
The pH value of 1-pyrroline solution in water as a function of concentration (**a**), and the signal intensity of 1-pyrroline vapor above the 200 ppb aqueous solution measured as a function of solution pH value (**b**).

Fig. 5a shows the pH value of 1-pyrroline solution in distilled water as a function of 1-pyrroline concentration. Significant elevation of pH from ∼7 starts at ca. 100 ppm concentration and reaches the level of ∼10 at the concentration 100,000 ppm. The onset of pH elevation roughly correlates with the onset of [*T*_*s*_] (Fig. 3a). Therefore, we propose that the elevation of pH at high 1-pyrroline concentration is related to the increase of [*T*_*s*_]/[*M*_*s*_] ratio and the increased contribution of *T*_*s*_ species.

Fig. 5b shows the APCI-MS signal intensity of 1-pyrroline in 200 ppb aqueous solution as a function of solution pH value. The pH value was adjusted using PBS buffer, sodium hydroxide solution and hydrochloric acid solution. At 200 ppb only *M*_*s*_ is present in solution (Fig. 3a). The pH dependence displays a pronounced step-like shape: more than three-order increase in signal intensity is observed between pH = 6 and pH = 8. This change in intensity corresponds to the transition through the pKa of 1-pyrroline (6.8). 1-Pyrroline, being a weak base, easily binds a proton in water. The equilibrium ratio between neutral and protonated 1-pyrroline species in water is determined by the pH value of the solution. The higher the pH the lower is the degree of 1-pyrroline protonation. Since 1-pyrroline evaporates only in its neutral form, the volatility of 1-pyrroline increases with the pH value of the solution. The growth in 1-pyrroline volatility with pH (Figs. 5b and S6) is most pronounced around pH 6.8, which corresponds to the pKa of 1-pyrroline. At this pH value, the ratio between neutral and protonated 1-pyrroline species is equal to 1:1. Boddeker *et al*. reported similar effect of pH on the evaporative enrichment of phenol^18^. The authors found that phenolic enrichment depends on the pH value of solution, which determines the equilibrium ratio between undissociated phenol and phenolate ion. Only undissociated phenol evaporates, whereas phenolate ion does not evaporate.

### The comparison of relative *M*_*s*_ and *T*_*s*_ volatility

The assessment of 1-pyrroline volatility based on the amount of evaporative loss is complicated by the poor long-term chemical stability of 1-pyrroline solutions^13^ under air and high pH sensitivity^14^. For the unbiased comparison of *M*_*s*_ and *T*_*s*_ volatility we used aprotic solvent (DMSO) and vacuum conditions in order to eliminate the pH factor, accelerate evaporation and eliminate the possibility of reaction with air. Fig. 4b displays the relative loss of 1-pyrroline in DMSO as a function of concentration over 30 min evaporation assisted by vacuum pumping (−0.1 MPa) at room temperature. The observed relative loss of 1-pyrrolnie was ca. 80% at concentrations below 10,000 ppm. At higher concentrations the rate of evaporative loss showed decrease with 1-pyrroline concentration. The evaporative loss of 1-pyrroline at 200,000 ppm concentration over 30 min is equal to ca. 20%, which corresponds to ca. 7 times lower evaporation rate of 1-pyrroline than in diluted solution. This estimate is consistent with the 7-times lower relative proportion of *M*_*s*_ in 200,000 ppm solution than in diluted solutions measured by NMR (Fig. 3a). Apparently, the evaporative loss of 200,000 ppm solution is mainly caused by *M*_*s*_ species, and the decrease of evaporative loss with 1-pyrroline concentration occurs mainly due to the decreased relative concentration of *M*_*s*_. Our experiments thereby indicate that *M*_*s*_ is much more volatile than *T*_*s*_ in DMSO solution. The pure 1-pyrroline, which is 100% trimer based on NMR data, showed only ca. 1% evaporative loss under the same experimental conditions. Based on these results, we estimate that the evaporation rate of neat 1-pyrroline (100% *T*_*s*_) is ca. 160 times lower than the evaporation rate of diluted (<10,000 ppm) 1-pyrroline in DMSO (100% *M*_*s*_) under the same conditions.

### Stability of *T*_*g*_

Density functional theory calculations at 6–31 + G(d, p) level show that the free energy of *M*_*g*_ is 18.8 kcal/mol lower than that of *T*_*g*_, indicating that *T*_*g*_ is less stable than *M*_*g*_ in the gas phase from the view of thermodynamics. The results of our calculations are consistent with the earlier estimate of 20 kcal/mol by Wiberg *et al*.^12^. The equilibrium [*M*_*g*_] and [*T*_*g*_] are interconnected via the equilibrium constant *K*_*eq*_^(g)^ = [*M*_*g*_] ^3^/[*T*_*g*_] = e^−ΔG(g)/RT^ = 4.8 × 10^13^ (at 300 K). The estimate vapor pressure of neat 1-pyrroline material at 300 K (liquid) is 0.005 atm, as derived by extrapolating the earlier measured data on the vapor pressure of neat 1-pyrroline material at 318–339 K^12^. The partial pressure estimates of *M*_*g*_ and *T*_*g*_ for neat 1-pyrroline are: *P*_*M*_ ≈ 0.005 atm; *P*_*T*_ ≈ 10^−27^ atm, i.e., one liter of air contains on average ∼10^−5^ molecules of *T*_*g*_. In other words, *T*_*g*_ is not present in the 1-pyrroline vapor under equilibrium with solution at any physically significant amount. According to earlier studies, the olfactory threshold concentration of 1-pyrroline in water is ca. 20 ppb^1^. This concentration roughly corresponds to the partial 1-pyrroline vapor pressure *P*_*threshold*_ ∼ 10^−8^ atm, which is ca. 20 orders of magnitude higher than the estimated equilibrium vapor pressure of *T*_*g*_. Overall, our results indicate that *T*_*g*_ is extremely unlikely to have any contribution to the odor of 1-pyrroline under equilibrium conditions. The odor of 1-pyrroline is most probably entirely contributed by *M*_*g*_.

**Figure 6:**
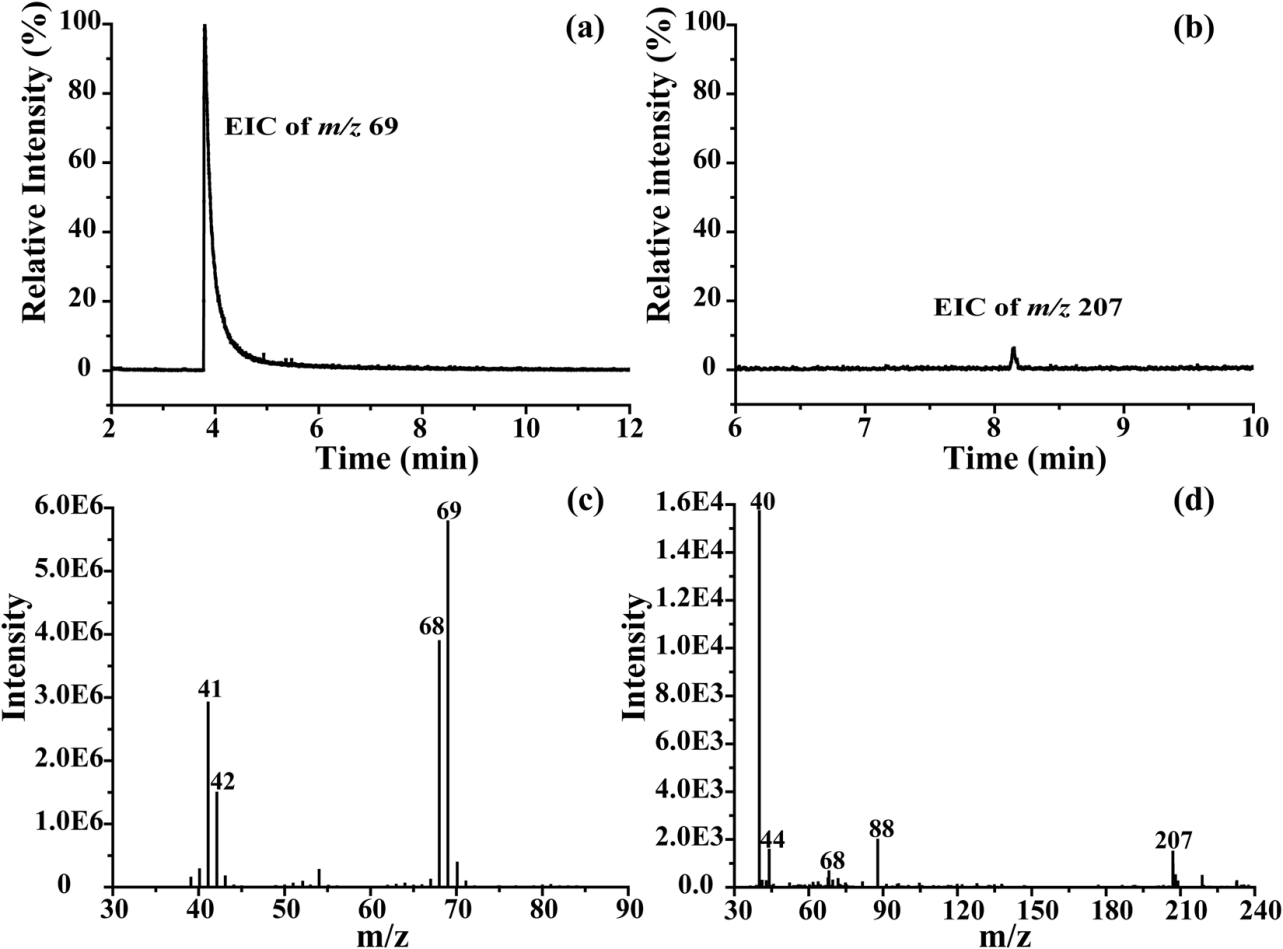
Extracted ion chromatograms of *m*/*z* 69 and *m*/*z* 207 signals and corresponding mass spectra at 4 min and 8.2 min elution time in GC-MS analysis of 1-pyrroline (100 ppm in CH_2_Cl_2_).

Edwards *et al*. reported that no trimer could be detected in the gas phase by IR and microwave spectroscopy of 1-pyrroline vapor^15^. Interestingly, our GC-MS analysis of 1-pyrroline in CH_2_Cl_2_, apart from the major *M*_*g*_ signal (*m*/*z* 69) at 4 min, also yielded the signal *m*/*z* 207 eluted at 8.2 min, corresponding to the radical cation of *T*_*g*_ (Fig. 6). This appears to be the first observation of 1-pyrroline trimer in the gas phase. The major characteristic ions at *m*/*z* 41, 42, 68, 69 were observed in the mass spectrum of 1-pyrroline monomer at 4 min, which are consistent with the earlier literature reports^19, 20^. As shown in Fig. 6, the *T*/*M* ratio in GC-MS experiments is greatly different from that in NMR experiments discussed above at the same solution concentration. A possible reason may be the different ionization efficiency and fragmentation behavior following ionization for the monomer and trimer. Another possible explanation is the partial decomposition of the trimer species, e.g., due to the high inlet temperature (150 °C). Therefore, the ratio of *m*/*z* 207 to *m*/*z* 69 in GC-MS cannot reflect the true *T*/*M* ratio in solution at the same solution concentration. However, the mere observation of *m*/*z* 207 (*T*_*g*_) in GC-MS is very curious as it indicates that under GC-MS conditions *T*_*g*_ appears to be rather stable. In fact, the trimer should be in gas state after GC injection and is somehow able to withstand very high temperature of the inlet (150 °C). Detailed studies may be required to evaluate the surprising stability of *T*_*g*_ revealed in GC-MS experiments and understand the factors responsible for such stability.

## Discussion

The article published in 1992 by Baker *et al*. presents by far the most detailed description of 1-pyrroline chemistry^13^. However, the current knowledge is yet far from complete. In fact, even the third-order equilibrium kinetics between *M*_*s*_ and *T*_*s*_ was postulated but has not been rigorously proven in the work by Baker *et al*. due to the lack of titration measurements. Our NMR data obtained at different dilution rates of 1-pyrroline in different solvents provides a clear-cut evidence for the third-order equilibrium kinetics (Fig. 3). The analysis of obtained concentration curves suggests that the relative stability of *T*_*s*_ in solution (ΔG ≈ −3.2 kcal/mol) is considerably higher than the earlier estimate by Baker *et al*. (ΔG ≈ −2 kcal/mol) obtained using NMR data from a single concentration point^13^. Another poorly understood aspect is the low chemical stability of 1-pyrroline in solution, e.g., in water or DMSO. The interpretation of experimental observations is greatly complicated by the equilibrium between the monomer and trimer 1-pyrroline species in the solution. Our experiments carried at different 1-pyrroline concentrations allowed independent consideration of the monomer and trimer chemistry and indicated that 1-pyrroline displays three distinct volatility behaviors: when present in neat form, in concentrated solution (>10,000 ppm), or in diluted solution (<1,000 ppm). Below we discuss these three cases separately.

1-Pyrroline in neat form is stable and exists purely in the trimeric form^13^. According to our experimental results, the volatility of neat 1-pyrroline is ca. 13 times lower than that of pure water. Our estimate is consistent with the earlier publications that reported the vapor pressure of 1-pyrroline at 300 K equal to 0.005 atm^12^, whereas the vapor pressure of water at 300 K is equal to 0.03 atm. The low volatility of neat 1-pyrroline is most probably related to the strong intermolecular bonding in neat material. Neat 1-pyrroline is a viscous liquid. The odor of neat 1-pyrroline is contributed by *M*_*g*_ produced by the spontaneous decomposition of *T*_*g*_.

1-Pyrroline in concentrated solution (ca. 10,000 ppm – 200,000 ppm) exists in equilibrium between the monomer and trimer states. The equilibrium obeys third-order kinetics. At room temperature *T*_*s*_ is more stable than *M*_*s*_ by ΔG ≈ 3.2 kcal/mol (in DMSO-*d*_6_). *M*_*s*_ in solution is ca. 100 times more volatile than *T*_*s*_. Accordingly, volatility of 1-pyrroline increases with the degree of dilution due to the increase of *M*_*s*_/*T*_*s*_ ratio. At 10,000 ppm the evaporation rate of 1-pyrroline from water exceeds the evaporation rate of water by ca. 7 times. Note that the uneven evaporation process does not alter the equilibrium *M*_*s*_/*T*_*s*_ ratio due to the rapid equilibrium kinetics: the evaporative loss of *M*_*s*_ from solution is continuously replenished to the equilibrium level by the rapid decomposition of *T*_*s*_ solutes^13^. The poor chemical stability of concentrated 1-pyrroline solutions discovered in earlier studies^13^ is probably related to the high chemical reactivity of *T*_*s*_ with the molecules of solvent and air. Baker *et al*. reported that 1-pyrroline in water solution (~200,000 ppm) stored at ambient conditions in 5-mm NMR sample tubes with loosely covered caps revealed approximately 20 additional ^13^C absorptions after three weeks of storage^13^. A rigid layer was formed at the surface of the degraded samples, suggesting that some type of polymerization occurred. We also observed the formation of a rigid upper-layer for 1-pyrroline in concentrated water solution. These observations indicate that the chemical instability of 1-pyrroline in concentrated solutions may be caused by the reactivity of 1-pyrroline with solvent and air. More research of these reactions is still needed to thoroughly understand the mechanism of 1-pyrroline chemical degradation.

1-Pyrroline in diluted solution (<1,000 ppm) exists mainly in the monomer state, and its volatility is independent on the degree of dilution (Fig. 4b). Earlier study by H. Poisel demonstrated that monomeric 1-pyrroline is relatively stable in dilute solution^21^. The higher chemical stability of diluted 1-pyrroline solutions (i.e. in the absence of trimer) suggests that the reactivity of concentrated 1-pyrroline solutions is mainly accounted by the trimer species. The study of 1-pyrroline reactivity, in particular ambient condensation in water, is a subject for further research.

Of particular interest is the step-wise dependence of 1-pyrroline volatility on pH (Fig. 5b). The abrupt increase of volatility corresponds to the transition through the pKa of 1-pyrroline (6.8). At pH < 6.8, 1-pyrroline is mostly protonated and displays lower volatility. At pH > 6.8, 1-pyrroline is mostly neutral and displays more than three-order of magnitude stronger volatility. While pKa for the majority of known nitrogenous volatile bases pKa is around 10 or 11^1^, the neutral pKa value of 1-pyrroline is rather unusual. Therefore, even minor pH alterations around neutral pH will be the cause of pronounced variation in odor strength. This may be an important evolutionary factor allowing living organisms to regulate the odor strength from very weak to very strong with minimal changes in the physiological pH.

## Methods

### Synthesis of 1-pyrroline

Several methods for the synthesis of 1-pyrroline have been introduced in earlier reports, such as silver (I)-catalyzed oxidation of pyrrolidine^22–24^, acid hydrolysis of 4-aminobutanal diethyl acetal^25, 26^. In our study, 1-pyrroline was synthesized according to the method described by Ogawa *et al*.^24^ based on easily available raw materials (silver and pyrrolidine) and was chosen for its rapidity and simplicity. Briefly, in a round-bottom flask covered with aluminum foil, a catalytic amount of AgNO_3_ (Shanghai Aladdin biochemical Polytron Technologies Inc, purity >99%; 0.37 mmol, 64 mg) was first added to a solution of pyrrolidine (Shanghai Aladdin biochemical Polytron Technologies Inc, purity >99%, 75 mmol, 5.33 g, 6.2 mL) in water (75 mL), after which NaOH pellets were added (0.15 mol, 6 g). To this mixture, a 25% aqueous solution of Na_2_S_2_O_8_ (75 mmol, 17.8 g, 70 mL) was added dropwise at ice bath. The reaction mixture was stirred for 3 hours at ambient temperature and was then extracted with CH_2_Cl_2_ (100 mL) and saturated NaCl water solution (50 mL) three times. After drying over Na_2_SO_4_ and removal of the solvent, the mixture was dissolved in ethyl ether and filtered through the plug of neutral alumina and was evaporated to dryness in vacuum. The resulting transparent viscous oil was used for NMR and MS analysis.

### NMR analysis

NMR spectra were measured on a Bruker DRX 500 Avance (1H, 500.13 MHz and 13C, 125.77 MHz) spectrometer at 298 K with tetramethylsilane (TMS) as the internal standard. Standard Bruker automated acquisition programs were used for all experiments. All one-dimensional spectra were composed by 64 K data points. The spectral width for ^1^H was set at 6024 Hz and for ^13^C at 18519 Hz with acquisition times of 5.439 and 1.769 sec, respectively. The resolution of the spectra was 0.565 Hz per point for ^13^C and 0.184 Hz per point for ^1^H. The numbers of scans for ^1^H and ^13^C were 16 and 1024, respectively. The following abbreviations were used to explain the multiplicities in Fig. 2: s = singlet, d = doublet, t = triplet, q = quartet, m = multiplet. Chemical shifts were assigned relative to TMS. Coupling constants (J) are reported in Hertz (Hz). The spectra and assignments were obtained with neat sample dissolved in different solutions (CDCl_3_/DMSO-*d*_6_/D_2_O) with different concentrations.

### Ambient MS analysis of 1-pyrroline vapor

Ambient MS^27, 28^ analysis of 1-pyrroline vapor was done on commercial ion trap mass spectrometer (LTQ-XL, Thermo Scientific, San Jose, CA, USA) using home-made corona discharge ionization^29^ and extractive electrospray ionization (EESI) sources as detailed in our earlier studies^30–32^. The headspace volatiles of 1-pyrroline solution (8 mL) in a 10 mL centrifuge tube (Solarbio, Beijing, China) were continuously transferred into ionization region via plastic tubing (ID 0.5 mm) assisted by nitrogen gas flow (0.1 MPa, 1 L/min). Ionizing voltage in the both ionization approaches was + 4.5 kV. Pure water and DMSO were used as solvent to prepare working solutions. The angle between the discharge needle and the outer tubing was 30 °C. The distance from the tip of the ion probe to the inlet of the MS capillary was 6mm. Each sample was analyzed in three replicates and collected for 30 s in the *m*/*z* range 15–300. The reference pure water or DMSO samples without 1-pyrroline were analyzed for background correction. The reference solutions with known 1-pyrroline concentration were analyzed under identical experimental conditions to construct the calibration curve for quantification purposes.

### GC-MS analysis

GC-MS analysis was performed on an Agilent triple quadrupole system (7890 B/7000C) equipped with an Agilent 7693 auto-sampler. The chromatographic separation was done on an HP-5 MS capillary column (30 m × 0.25 mm, film thickness 0.25 μm, Agilent Technologies, USA). The front inlet was kept at 150 °C in the split mode. The GC oven temperature program was as follows: initial column temperature 30 °C, held for 5 min, then programmed to 70 °C at a rate of 10 °C per minute and held for 2 min and finally programmed to 250 °C at a rate of 30 °C per minute and held at 250 °C for 3 min. Helium was used as the carrier gas at a constant flow rate of 1.0 mL/min. The injection volume was 1 μL. The MS detector was used in the electron ionization (EI) mode with an ionization voltage of 70 eV. The ion source temperature was at 230 °C. The transfer line was at 280 °C. The spectra were collected over the *m*/*z* range 30–300.

### Structure calculations

Structure calculations were done using Gaussian 03 software^33^. The geometries of the target species were optimized using the density functional theory (DFT) method at the B3LYP/6–31 + G(d,p) level. The optimized structures were identified as a true minimum in energy by the absence of imaginary frequencies. Vibrational frequencies of all the key species were calculated at the same level of theory. The optimized structures were displayed by the Gauss View (05) software. The energies discussed here are the sum of electronic and thermal free energy. Calculation parameters and results are shown in output files in Supplementary Material.

## Electronic supplementary material

Supplementary information
