# Supplementary material for: On the chemistry of 1-pyrroline in solution and in the gas phase
Source: Sci Rep. 2017 Aug 9;7:7675. doi: 10.1038/s41598-017-08217-1 (PMC5550421; doi:10.1038/s41598-017-08217-1)
Supplement: Supplementary file 1 — Supplementary information [file 41598_2017_8217_MOESM1_ESM.doc]

**On the chemistry of 1-pyrroline in solution and in the gas phase**

Xiaoping Zhang, Konstantin Chingin,^*^ Dacai Zhong, Juchao Liang, Yongzhong Ouyang, and Huanwen Chen

Jiangxi Key Laboratory for Mass Spectrometry and Instrumentation, East China University of Technology, Nanchang 330013 P.R. China

**SUPPLEMENTARY INFORMATION**

**Figure S1.** ^1^H-NMR spectrum of 1-pyrroline solution in D_2_O (10,000 ppm) displaying the chemical shift assignments for the monomer and trimer.

**Figure S2.** ^1^H-NMR spectrum of 1-pyrroline solution in CDCl_3_ (10,000 ppm) displaying the chemical shift assignments for the monomer and trimer.

**Figure S3.** The relative proportion of *T_s_* and *M_s_* in CDCl_3_ solution (a) and D_2_O (b) as a function of 1-pyrroline concentration.

**Figure S4.** APCI-MS spectrum of 1-pyrroline solution in H_2_O (10,000 ppm).

**Figure S5.** The linear range of 1-pyrroline APCI-MS signal (*m/z* 70) in the concentration range from 10 to 400 ppb in H_2_O (a) and from 1 to 100 ppb in DMSO (b).

**Figure S6.** APCI-MS spectra of 1-pyrroline in H_2_O solvent at different pH value. (a) pH 3, (b) pH 5, (c) pH 7, (d) pH 9, (e) pH 11.

**NMR data**

Monomer: ^1^H NMR (500 MHz, CDCl_3_) δ 7.62 (s, 1H), 3.82-3.88 (m, 2H), 2.51-2.57 (t, 2H), 1.70-1.90 (m, 2H); Trimer: ^1^H NMR (500 MHz, CDCl_3_) δ 1.70-1.90 (m, 12H), 2.90-3.10 (m, 6H), 2.29-2.37 (m, 3H). Monomer: ^1^H NMR (500 MHz, D_2_O) δ 7.49 (s, 1H), 3.54-3.62 (m, 2H), 2.37-2.44 (t, 2H), 1.45-1.85 (m, 2H); Trimer: ^1^H NMR (500 MHz, D_2_O) δ 2.99-3.05 (t, 3H), 2.70-2.78 (m, 3H), 2.22-2.30 (dt, 3H), 1.45-1.85 (m, 12H).

**
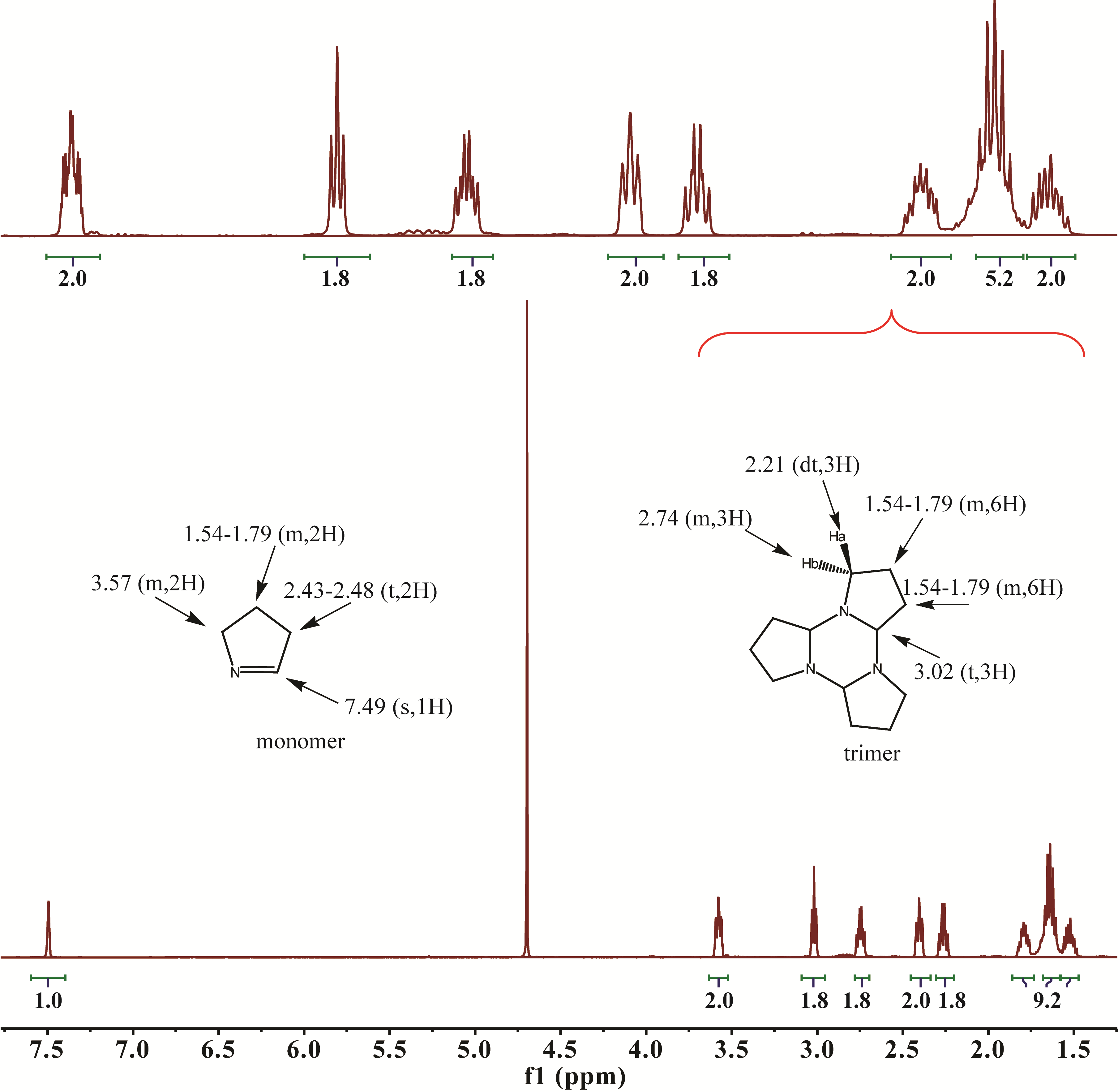
**

**Figure S1.** ^1^H-NMR spectrum of 1-pyrroline solution in D_2_O (10,000 ppm) displaying the chemical shift assignments for the monomer and trimer.


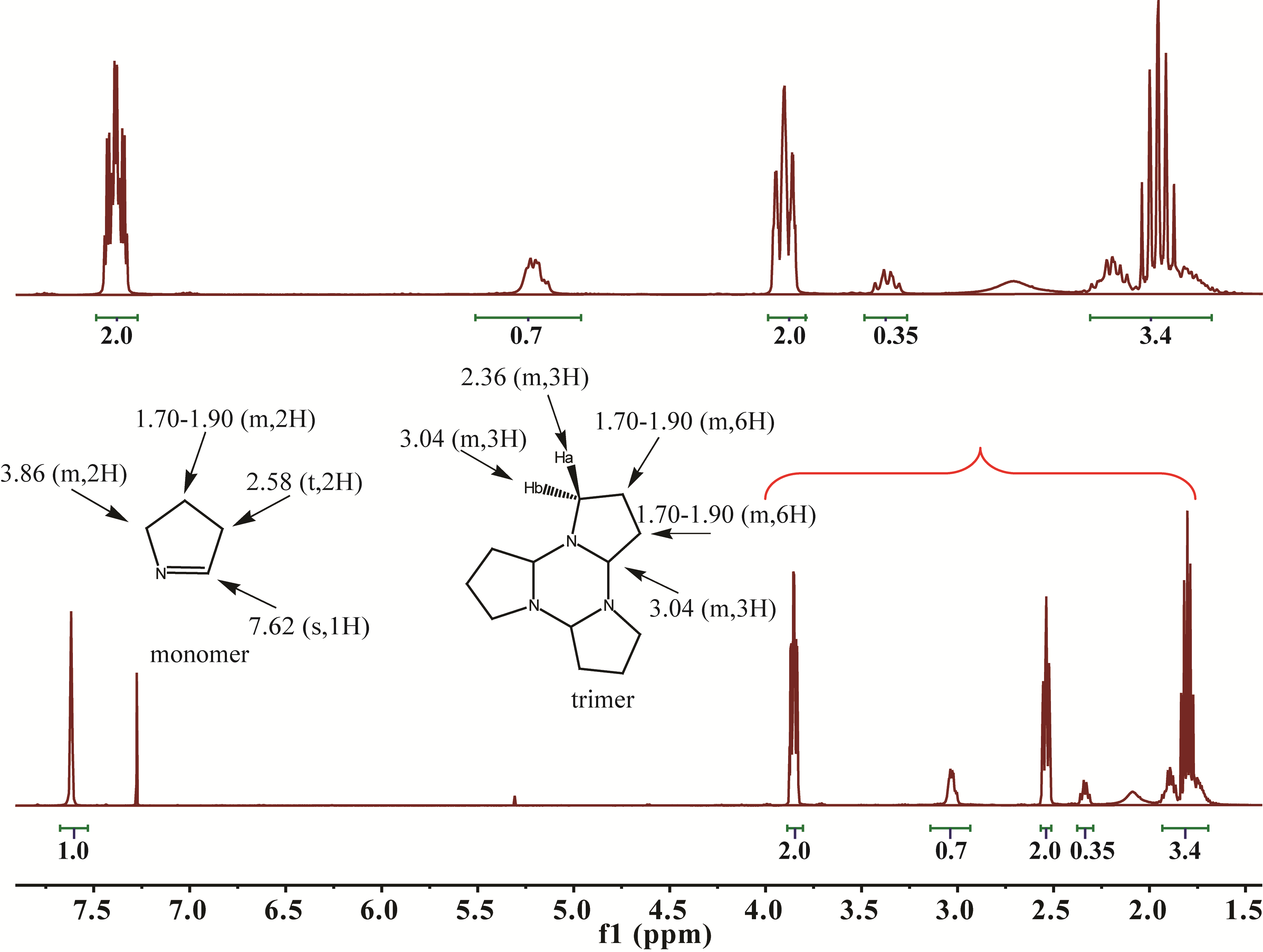


**Figure S2.** ^1^H-NMR spectrum of 1-pyrroline solution in CDCl_3_ (10,000 ppm) displaying the chemical shift assignments for the monomer and trimer.


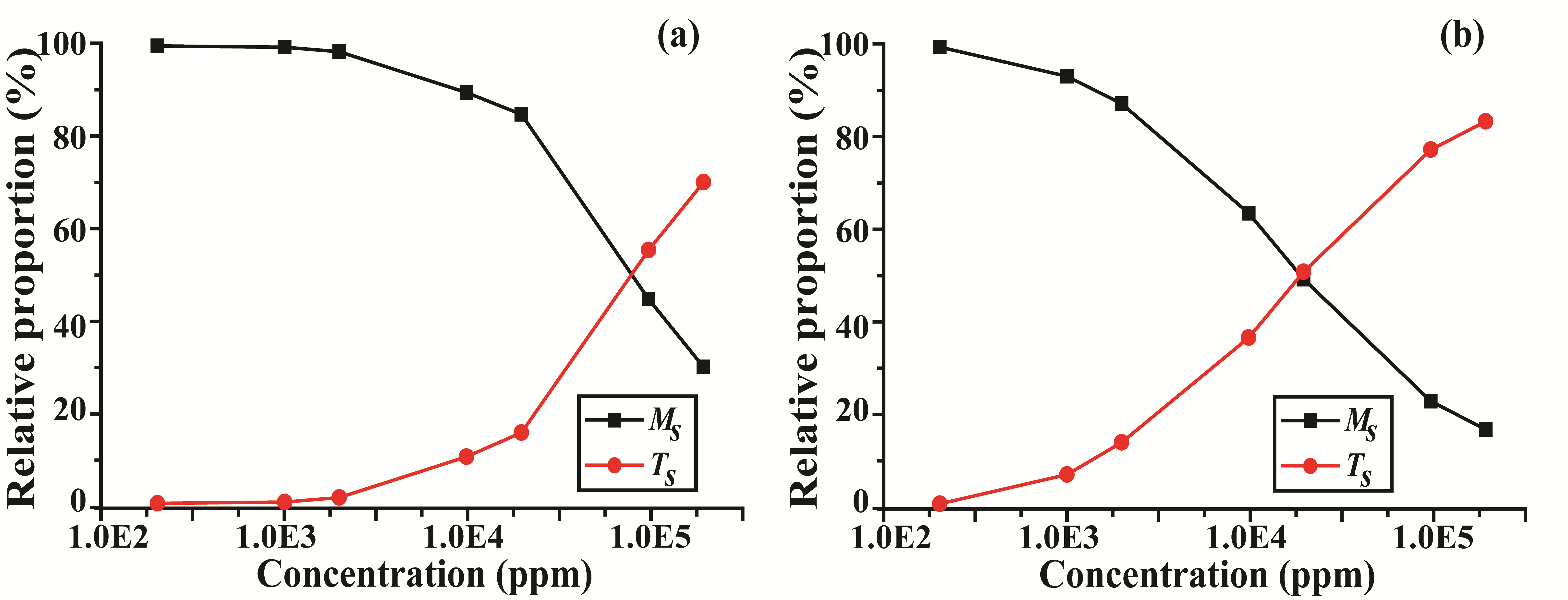


**Figure S3.** The relative proportion of *T_s_* and *M_s_* in CDCl_3_ solution (a) and D_2_O (b) as a function of 1-pyrroline concentration.

**
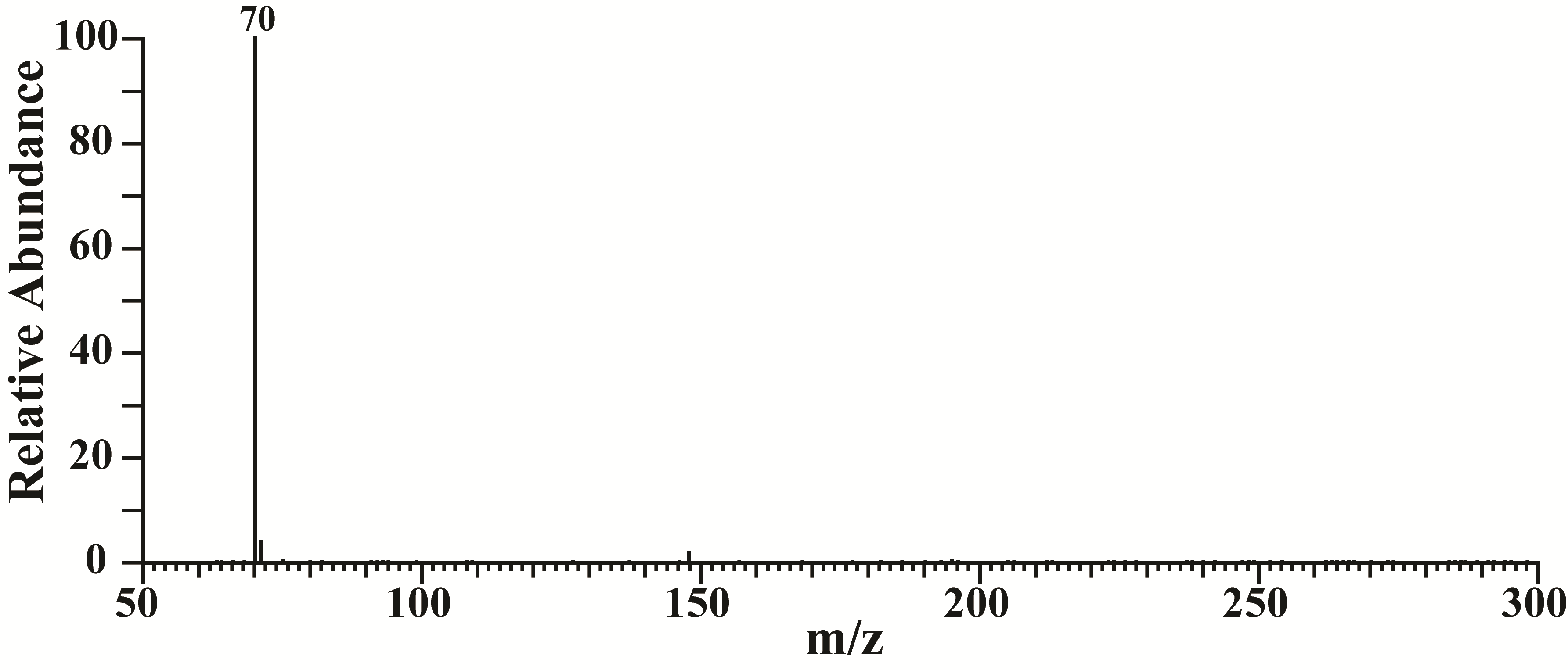
**

**Figure S4.** APCI-MS spectrum of 1-pyrroline solution in H_2_O (10,000 ppm).


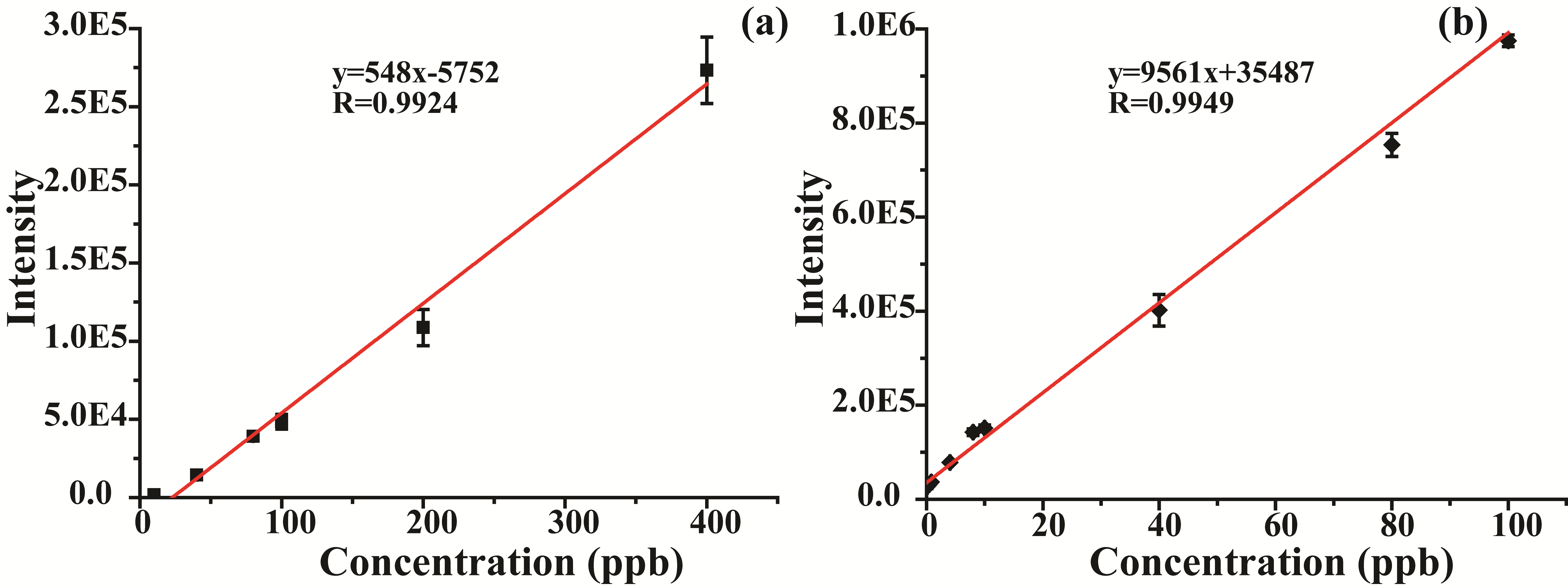


**Figure S5.** The linear range of 1-pyrroline APCI-MS signal (*m/z* 70) in the concentration range from 10 to 400 ppb in H_2_O (a) and from 1 to 100 ppb in DMSO (b).


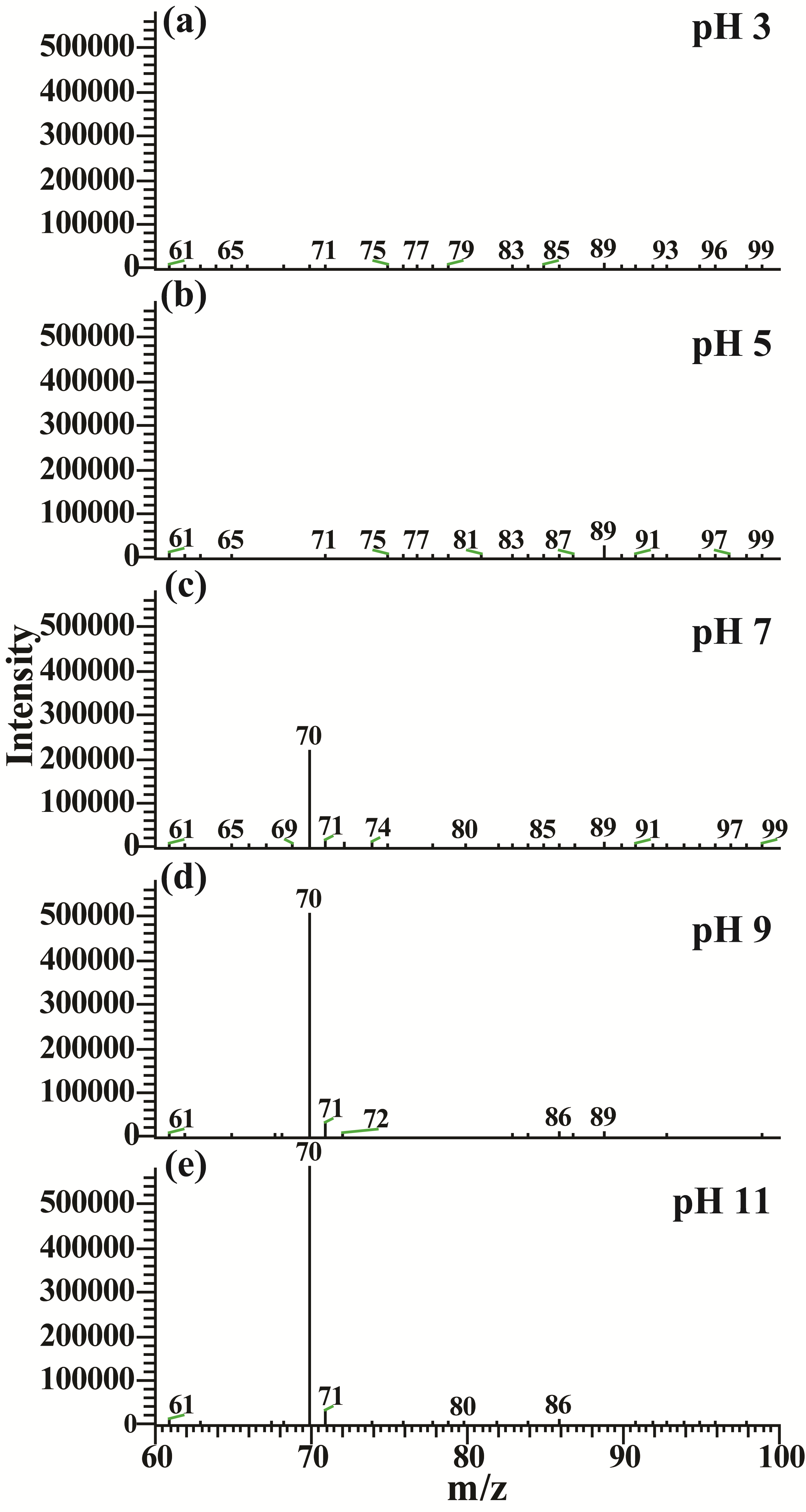


**Figure S6.** APCI-MS spectra of 1-pyrroline in H_2_O solvent at different pH value. (a) pH 3, (b) pH 5, (c) pH 7, (d) pH 9, (e) pH 11.

**Quantum chemistry calculations of 1-pyrroline energy**

**
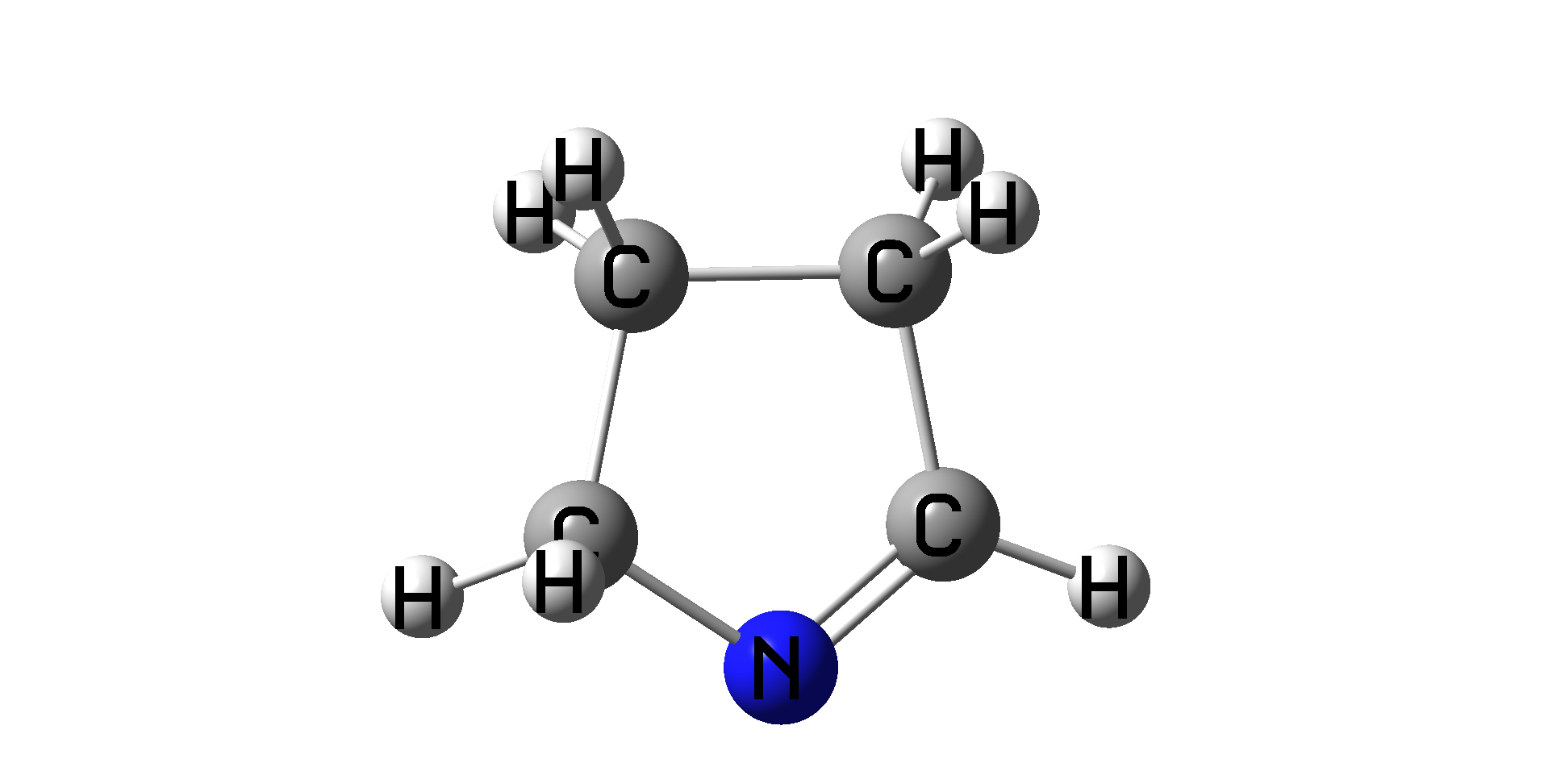
**

Zero-point correction= 0.105179 (Hartree/Particle)

Thermal correction to Energy= 0.109730

Thermal correction to Enthalpy= 0.110674

Thermal correction to Gibbs Free Energy= 0.077725

Sum of electronic and zero-point Energies= -211.285018

Sum of electronic and thermal Energies= -211.280467

Sum of electronic and thermal Enthalpies= -211.279523

Sum of electronic and thermal Free Energies= -211.312472 ---------------------------------------------------------------------

Center Atomic Atomic Coordinates (Angstroms)

Number Number Type X Y Z

---------------------------------------------------------------------

1 6 0 0.003751 -0.030856 0.008284

2 6 0 0.001837 0.045539 1.559816

3 6 0 1.503311 -0.043545 1.921504

4 6 0 2.152208 0.227937 0.579404

5 7 0 1.384025 0.240378 -0.438693

6 1 0 -0.676286 0.688039 -0.459982

7 1 0 -0.411697 1.003036 1.892711

8 1 0 1.796529 -1.039044 2.283704

9 1 0 3.225319 0.382755 0.461441

10 1 0 1.816513 0.674466 2.687788

11 1 0 -0.594003 -0.747475 2.019497

12 1 0 -0.280132 -1.023990 -0.364058

---------------------------------------------------------------------


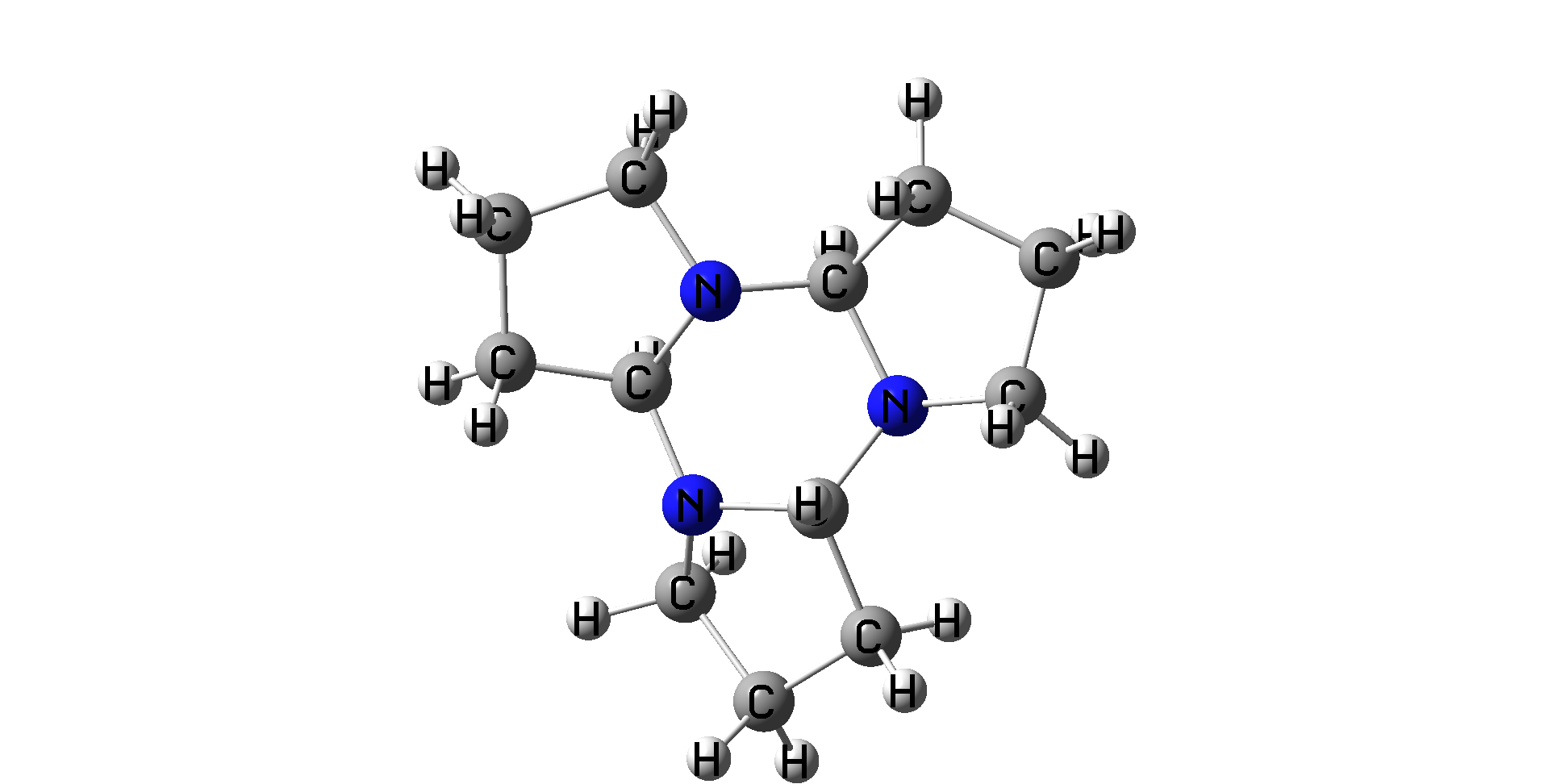


Zero-point correction= 0.327017 (Hartree/Particle)

Thermal correction to Energy= 0.339705

Thermal correction to Enthalpy= 0.340649

Thermal correction to Gibbs Free Energy= 0.287532

Sum of electronic and zero-point Energies= -633.867951

Sum of electronic and thermal Energies= -633.855263

Sum of electronic and thermal Enthalpies= -633.854319

Sum of electronic and thermal Free Energies= -633.907436

---------------------------------------------------------------------

Center Atomic Atomic Coordinates (Angstroms)

Number Number Type X Y Z

---------------------------------------------------------------------

1 6 0 0.000000 0.000000 0.000000

2 6 0 0.000000 0.000000 2.395978

3 6 0 2.179052 0.000000 1.229456

4 6 0 2.069883 -0.005944 -1.225626

5 6 0 0.913129 0.176743 -2.213785

6 6 0 -0.183220 0.769996 -1.317542

7 1 0 2.602490 0.955451 -1.093498

8 1 0 0.598983 -0.792702 -2.618228

9 1 0 0.008010 1.835049 -1.138208

10 6 0 -0.638909 0.828350 3.528892

11 6 0 -1.715176 1.161997 1.349706

12 6 0 -1.818063 1.569425 2.837582

13 1 0 -1.710009 2.652158 2.947424

14 1 0 0.106496 1.530296 3.912067

15 1 0 -2.391339 0.310095 1.135284

16 6 0 3.468683 -0.846687 1.405183

17 6 0 1.887052 -1.440716 3.114500

18 6 0 3.391545 -1.422691 2.835342

19 1 0 3.849392 -2.413228 2.916452

20 1 0 1.411613 -2.337944 2.672463

21 1 0 4.374468 -0.255501 1.240295

22 7 0 -0.315389 0.769549 1.193193

23 7 0 1.426262 -0.429897 0.030895

24 7 0 1.435718 -0.204796 2.477074

25 1 0 3.455053 -1.656280 0.669434

26 1 0 3.890456 -0.758822 3.549316

27 1 0 1.649747 -1.431066 4.184437

28 1 0 2.805967 -0.745093 -1.560862

29 1 0 1.179245 0.822811 -3.055680

30 1 0 -1.190966 0.665191 -1.731168

31 1 0 -1.972935 1.972662 0.661700

32 1 0 -2.791600 1.295095 3.253929

33 1 0 -0.965460 0.194007 4.358344

34 1 0 -0.511810 -0.993640 2.343062

35 1 0 -0.634238 -0.913638 -0.013958

36 1 0 2.419811 1.073076 1.134356

---------------------------------------------------------------------
